# High-intensity exercise during chemotherapy induces beneficial effects 12 months into breast cancer survivorship

**DOI:** 10.1007/s11764-019-00747-z

**Published:** 2019-03-25

**Authors:** Sara Mijwel, Anna Jervaeus, Kate A. Bolam, Jessica Norrbom, Jonas Bergh, Helene Rundqvist, Yvonne Wengström

**Affiliations:** 1 Department of Neurobiology, Care Sciences and Society, Karolinska Institutet, Stockholm, Sweden; 2 Cancer Theme, Karolinska University Hospital, Stockholm, Sweden; 3 Department of Physiology and Pharmacology, Karolinska Institutet, Stockholm, Sweden; 4 Department of Oncology and Pathology Cancer Center Karolinska, Karolinska Institutet, Stockholm, Sweden; 5 Department of Cell and Molecular Biology, Karolinska Institutet, Stockholm, Sweden

**Keywords:** Randomized controlled trial, Cancer-related fatigue, Breast cancer, High-intensity interval training, Cancer survivorship

## Abstract

**Purpose:**

Whether the benefits of exercise during chemotherapy continue into survivorship is not well-known. Here, the aim was to examine the effects of two exercise interventions on self-reported health-related and objectively measured physiological outcomes 12 months following commencement of chemotherapy.

**Methods:**

Two hundred and forty women with breast cancer stage I–IIIa were randomized to 16 weeks of high-intensity aerobic interval training combined with either resistance training (RT-HIIT), or moderate-intensity aerobic training (AT-HIIT), or to usual care (UC). Primary outcome: cancer-related fatigue (CRF); secondary outcomes: quality of life (QoL), symptom burden, muscle strength, cardiorespiratory-fitness, body mass, and return to work.

**Results:**

Compared to UC, both RT-HIIT and AT-HIIT significantly counteracted increases in total CRF (ES = − 0.34; ES = − 0.10), daily life CRF (ES=-0.76; ES=-0.50, and affective CRF (ES=-0.60; ES=-0.39). Both RT-HIIT and AT-HIIT reported significantly lower total symptoms (ES = − 0.46, ES = − 0.46), and displayed gains in lower limb (ES = 0.73; ES = 1.03) and handgrip muscle strength (surgery side ES = 0.70, ES = 0.71; non-surgery side ES = 0.57, ES = 0.59). AT-HIIT displayed significant reductions in body mass (ES = − 0.24), improved QoL: role (ES = 0.33) and emotional functioning (ES = 0.40), and a larger proportion had returned to work (*p* = 0.02) vs UC.

**Conclusion:**

These findings emphasize the beneficial effects of supervised high-intensity exercise during chemotherapy to improve the health and to reduce societal costs associated with prolonged sick leave for patients with breast cancer several months following chemotherapy.

**Implications for Cancer Survivors:**

These findings provide important information with substantial positive consequences for breast cancer survivorship. High-intensity exercise programs during chemotherapy and support to maintain physical activity can be a powerful strategy to manage or prevent many of the short- and long-term adverse effects of treatment for the increasing cohort of cancer survivors.

## Introduction

Advanced treatment strategies, which lead to improved survival for patients diagnosed with early stage breast cancer [1], also mean that more patients are at risk of suffering from toxic effects of chemotherapy regimens such as persistent debilitating symptoms that remain into survivorship [2]. During the survivorship period, which we here refer to as the period following primary breast cancer treatment in accordance with the physical activity and cancer control framework [3], cancer-related fatigue (CRF) has been reported to be one of the most debilitating symptoms and can persist for years [4]. Moreover, CRF has been shown to be a major barrier to perform physical activity (PA), and affects physical and psychosocial well-being and ability to work [5]. Women who have undergone chemotherapy for breast cancer experience declines in cardiorespiratory fitness and muscular strength [6–8]. The results from the original 16-week OptiTrain study [9, 10] showed beneficial effects of high-intensity interval training, particularly in combination with resistance exercise, on cancer-related fatigue, symptoms, muscle strength, cardiorespiratory fitness, and body mass. Our findings are in line with a number of other trials [11]; however, few trials have reported on long-term effects of exercise during chemotherapy [12–17]. Here, the long-term effects of two different supervised exercise programs during chemotherapy were investigated with focus on objectively measured physiological and self-reported health-related outcomes. During the follow-up period, an effort was also made to support the participants to maintain physical exercise. At 12 months, compared to baseline, we hypothesized that both exercise groups would display sustained levels for health-related and physiological outcomes whereas the control group would show declined levels. The reason why sustained effects were expected were hypothesized to be due to an increased knowledge and awareness of the participants’ physical capabilities that were gained from the supervised exercise sessions and due to the support to maintain physical activity during the follow-up period. The aim of this study was to investigate and compare the effects of the OptiTrain exercise interventions on the primary outcome: cancer-related fatigue and secondary outcomes: health-related quality of life (HRQoL), symptoms, physiological outcomes, and return to work 12 months following the commencement of chemotherapy in women with breast cancer, i.e. 9 months after completion of the intervention.

## Methods

### Participants and procedures

The methods of the OptiTrain randomized controlled trial (NCT02522260, Optimal Training Women with Breast Cancer (OptiTrain), www.clinicaltrials.gov) have been reported elsewhere [9, 10]. In brief, participants were recruited from two different oncology clinics in Stockholm, Sweden, from March 2013 to July 2016. Eligibility criteria included women (i) aged 18–70 years, (ii) diagnosed with I–IIIa stage breast cancer, and (iii) planned to receive adjuvant chemotherapy. Participants were randomized to 16 weeks of resistance training combined with high-intensity interval training (RT-HIIT), moderate-intensity aerobic training combined with high-intensity interval training (AT-HIIT), or usual care (UC). The intervention groups (RT-HIIT and AT-HIIT) commenced the exercise training 3 days after the second chemotherapy session and ended the intervention 3 weeks following the last chemotherapy session. Details regarding the randomization process and blinding have been explained elsewhere [10]. Ethical approval was obtained from the Regional Ethical Review Board in Stockholm Sweden (Dnr 2012/1347-31/1, 2012/1347-31/2, 2013/7632-32, 2014/408-32) and was conducted in accordance with the Helsinki Declaration. All participants gave written informed consent.

**Fig. 1:**
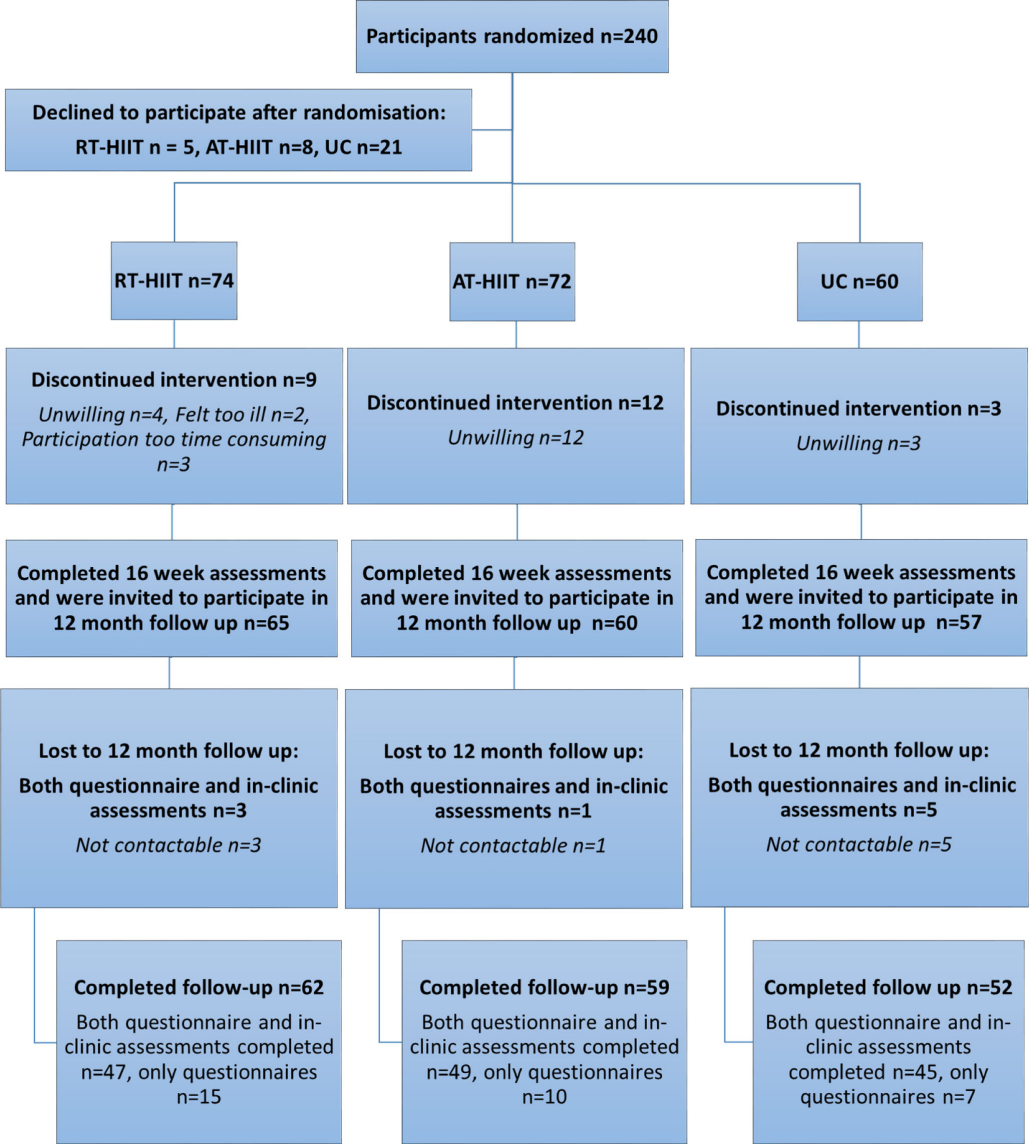
Participant flow through the 12 month follow-up study

The participant flow during and following the exercise trial is shown in Fig. 1. Participants were contacted by phone or e-mail for follow-up measurements, which took place 12 months following baseline assessments. The primary outcome was cancer-related fatigue and secondary outcomes included muscle strength, cardiorespiratory fitness, body mass, HRQoL, symptoms, and return to work. If participants agreed to participate, reassessments took place in the in-clinic gym where muscular strength (isometric mid-thigh pull and handgrip), estimated cardiorespiratory fitness (Åstrand-Rhyming submaximal cycle test), and body mass were measured as previously described [10]. Questionnaires were sent by post or electronically to those with e-mail addresses and consisted of the revised Piper Fatigue Scale (PFS) [18, 19], European Organization for Research and Cancer Treatment Quality of Life Questionnaire (EORTC-QLQ-C30) [20], and Memorial Symptom Assessment Scale (MSAS) [21]. A question regarding how much sick leave the participants were taking at 12 months was included with five possible options: 0%, 25%, 50%, 75%, or 100%, as well as a self-reported single item question of the participants’ PA levels allowing categorization of participants as not meeting the exercise recommendations of at least 150 min of moderate-intensity exercise per week [22, 23] or meeting exercise recommendations at baseline, 16 weeks, and at 12 months. Objectively measured activity patterns were assessed only at baseline by an accelerometer (GT3X ActiGraph® Corp, Pensacola, FL, USA). Further details regarding methods and analysis have been explained elsewhere [9]. For the follow-up, procedures of the mentioned outcomes were identical to the original study [9, 10].

### Exercise training interventions

The OptiTrain exercise protocol has been described previously [9, 24]. The exercise groups undertook the supervised exercise sessions in an exercise clinic twice weekly for 16 weeks and the session duration was approximately 60 min. HIIT was combined either with resistance training or incorporated as part of an overall endurance training session (AT-HIIT) in order to obtain similar exercise durations in both groups. In brief, the RT-HIIT group performed high-load resistance exercises targeting the major muscle groups consisting of two to three sets of 8–12 repetitions at an initial intensity of 70% of their estimated one repetition maximum (1-RM), progressing to 80% of 1-RM. The RT-HIIT sessions concluded with 3 × 3-min bouts of high-intensity interval exercise on a cycle ergometer interspersed with 1 min of recovery. The AT-HIIT group initiated each session with 20 min of moderate-intensity continuous aerobic exercise followed by the same high-intensity interval exercise as in RT-HIIT. The UC group was given written information about physical activity at the initiation of the intervention period about exercise recommendations for patients with cancer according to the American College of Sports Medicine guidelines [23].

### Motivational seminars and exercise sessions to maintain physical exercise adherence

Directly after the completion of the 16-week exercise program, participants in the exercise groups were offered a written, basic, PA on prescription by nurses involved in the exercise trial. The prescription was based on the text book Physical Activity in the Prevention and Treatment of Disease for evidence-based prescriptions [25]. During the follow-up, in collaboration with a national gym organization Friskis & Svettis, these participants were offered a one-on-one exercise counseling session with a professional health educator which they were able to use at any time during the follow-up period and were offered the opportunity to purchase gym cards at a reduced rate. Participants were also invited to an additional two to three motivational exercise sessions per year during the follow-up period (seven in total during the follow-up period between 2014 and 2017) that included motivational presentations and physical exercise demonstrations organized by the research team in collaboration with Friskis & Svettis. The UC group were not given any advice other than printed written information about exercise recommendations for patients with cancer according to the American College of Sports Medicine guidelines at the initiation of the intervention [22].

### Statistical analysis

The calculation of the sample size was based on the primary outcome CRF and has previously been reported [9]. Variables were visually checked for normality through QQ-plots and skewness. Non-normally distributed data were log-transformed. Baseline and 12-month medical and demographic characteristics were summarized using descriptive statistics (Table 1). Exact χ^2^ tests were used for categorical variables and a one-way ANOVA for continuous data. Linear mixed model using unstructured covariance model was used to assess differences between groups at the 12-month follow-up, adjusted for baseline values of the outcome. For the primary outcome, the model was also adjusted for tumor receptor and menopausal status. Group-by-time interaction was included as a fixed effect. Since the model includes all available data (i.e., baseline, 16-weeks, and 12 months), intention to treat analysis was possible without imputing data. Data available from 16 weeks were included in the model but only presented as mean ± SD in Tables 2, 3, 4, and 5. Adjustment for multiple groupwise comparisons were performed by using Bonferroni post hoc corrections. Effect sizes were calculated as the mean pre-post change in the treatment group minus the mean pre-post change in the control group, divided by the pooled pretest standard deviation [26]. Pooling only the pretest standard deviation has been shown to provide an unbiased estimate of the effect size [26]. According to Cohen’s guidelines, effect sizes with scores of 0.2–0.5, 0.5–0.8, and > 0.8 were considered small, medium, and large effects, respectively [27]. Pearson’s coefficient of correlation (*r*) was used to evaluate the association between changes in physiological outcomes and changes in CRF. The statistical software used was SPSS version 24 (IBM corporation, Chicago, IL, USA). All tests were two-tailed and significance level was set at *p* < 0.05.

**Table 1:** Participant characteristics at baseline

	RT-HIIT *n* = 74	AT-HIIT *n* = 72	UC *n* = 60	
	Mean ± SD	Mean ± SD	Mean ± SD	*p* value*
Age (years)	52.7 ± 10.3	54.4 ± 10.3	52.6 ± 10.2	0.89
Body mass (kg)	68.7 ± 11.3	67.7 ± 13.0	69.1 ± 11.0	0.37
Height (cm)	165.7 ± 6.7	165.3 ± 6.6	166.4 ± 7.0	0.76
SED (% of daily wear time)	63.7 ± 7.7	65.6 ± 6.2	66.6 ± 7.2	0.28
MVPA (% of daily wear time)	9.6 ± 2.8	8.3 ± 2.8	8.5 ± 4.3	0.14
	%	%	%	*p* value†
Married or partnered	60.6	59.7	69.5	0.81
University completed	67.6	64.7	66.0	1.00
Postmenopausal women	51.4	63.9	61.7	0.39
Tumor receptor status				0.15
Triple negative	14.9	11.0	16.7	
HER2+, ER±	21.6	30.2	20.0	
HER2−, ER+	62.2	58.9	61.6	
HER2−, ER−	1.4	0.0	1.7	
Hormone therapy	85.7	84.4	70.5	0.40
Chemotherapy received at baseline				0.98
Taxane-based therapy	40.6	37.0	41.7	
Anthracycline-based therapy	59.4	63.0	58.3	

*SD* standard deviation, *RT-HIIT* resistance and high-intensity interval training, *AT-HIIT* moderate-intensity aerobic and high-intensity interval training, *UC* usual care

*One-way ANOVA

^†^Exact *χ*^2^ test

**Table 2:** Effects of the OptiTrain intervention during chemotherapy on cancer-related fatigue at 12 months

						Adjusted group differences in mean change from baseline to 12 months (95% CI)				
		Baseline	16 weeks	12 months		Mean change	Lower bound 95% CI	Upper bound 95% CI	*p* value	ES
PFS questionnaire		Mean ± SD	Mean ± SD	Mean ± SD						
Behavior/daily life CRF	RT-HIIT	3.10 ± 3.39^ɸ^	3.01 ± 3.31	2.34 ± 2.68	RT-HIIT vs UC	− 1.65	− 2.71	− 0.59	*0.001*	− 0.76
	AT-HIIT	1.87 ± 2.57	2.98 ± 2.89	2.16 ± 2.52	AT-HIIT vs UC	− 1.29	− 2.36	− 0.23	*0.011*	− 0.50
	UC	2.04 ± 2.79	4.02 ± 3.29	3.48 ± 3.13						
Emotional/affective CRF	RT-HIIT	3.28 ± 3.43	3.45 ± 3.33	3.12 ± 2.89	RT-HIIT vs UC	− 1.35	− 2.51	− 0.19	*0.017*	− 0.60
	AT-HIIT	2.37 ± 2.98	3.74 ± 3.23	3.02 ± 2.96	AT-HIIT vs UC	− 1.19	− 2.36	− 0.02	*0.045*	− 0.39
	UC	2.45 ± 3.01	4.24 ± 3.22	4.10 ± 3.15						
Sensory/physical CRF	RT-HIIT	3.24 ± 3.23	3.27 ± 3.19	3.04 ± 2.83	RT-HIIT vs UC	− 1.05	− 2.12	0.03	0.061	− 0.46
	AT-HIIT	2.27 ± 2.87	3.53 ± 3.15	2.88 ± 2.83	AT-HIIT vs UC	− 0.88	− 1.96	0.20	0.154	− 0.24
	UC	2.64 ± 3.15	4.29 ± 3.31	3.82 ± 3.07						
Cognitive CRF	RT-HIIT	2.79 ± 2.95	2.82 ± 2.84	2.77 ± 2.78	RT-HIIT vs UC	− 0.88	− 1.94	0.18	0.141	− 0.23
	AT-HIIT	1.88 ± 2.51	2.61 ± 2.39	2.24 ± 2.29	AT-HIIT vs UC	− 1.17	− 2.24	− 0.10	*0.028*	− 0.13
	UC	2.15 ± 2.70	3.47 ± 2.87	3.41 ± 2.90						
Total CRF	RT-HIIT	3.09 ± 3.17	3.12 ± 3.03	2.80 ± 2.64	RT-HIIT vs UC	− 1.23	− 2.25	− 0.21	*0.012*	− 0.34
	AT-HIIT	2.10 ± 2.63	3.18 ± 2.77	2.58 ± 2.55	AT-HIIT vs UC	− 1.11	− 2.14	− 0.09	*0.029*	− 0.10
	UC	2.30 ± 2.81	3.98 ± 3.05	3.68 ± 2.94						

*ES* effect size, *PFS* Piper fatigue scale, *CRF* cancer-related fatigue, *RT-HIIT* resistance and high-intensity interval training group, *AT-HIIT* moderate-intensity and high-intensity interval training group, *UC* usual care group

*Adjusted group differences based on all available data in the mixed-model analysis adjusted for baseline values, menopausal status, and tumor receptor status; *p* < 0.05 is highlighted in italics

†Significant difference at baseline between RT-HIIT and AT-HIIT

**Table 3:** Effects of the OptiTrain trial during chemotherapy on quality of life at 12 months

						Adjusted group differences in mean change from baseline to 12 months (95% CI)*				
		Baseline	16 weeks	12 months		Mean change	Lower bound 95% CI	Upper bound 95% CI	*p* value	ES
EORTC-QLQ-C30 questionnaire		Mean ± SD	Mean ± SD	Mean ± SD						
Global/quality of life	RT-HIIT	63.56 ± 24.97	63.85 ± 19.88	73.41 ± 20.57	RT-HIIT vs UC	3.56	− 3.81	10.93	0.734	0.30
	AT-HIIT	66.67 ± 20.90	63.75 ± 20.29	77.12 ± 13.54	AT-HIIT vs UC	6.11	− 1.30	13.52	0.143	0.36
	UC	68.84 ± 21.66	59.52 ± 19.62	71.72 ± 19.84						
Physical functioning	RT-HIIT	89.38 ± 14.67	85.88 ± 16.31	91.58 ± 13.77	RT-HIIT vs UC	3.68	− 2.13	9.49	0.383	0.26
	AT-HIIT	89.98 ± 11.42	85.86 ± 15.37	91.87 ± 13.55	AT-HIIT vs UC	4.24	− 1.59	10.06	0.241	0.27
	UC	88.25 ± 16.58	76.91 ± 20.22	86.40 ± 15.84						
Emotional functioning	RT-HIIT	67.85 ± 25.80	72.30 ± 22.92	75.00 ± 26.14	RT-HIIT vs UC	4.57	− 3.77	12.91	0.562	0.35
	AT-HIIT	74.42 ± 18.94	79.86 ± 16.19	81.40 ± 16.49	AT-HIIT vs UC	9.10	0.71	17.50	*0.029*	0.40
	UC	74.47 ± 24.32	69.35 ± 26.26	72.79 ± 26.41						
Role functioning	RT-HIIT	59.91 ± 34.72	70.83 ± 28.27	85.49 ± 22.07	RT-HIIT vs UC	7.55	− 2.01	17.10	0.173	0.46
	AT-HIIT	67.61 ± 30.55	71.39 ± 26.59	88.34 ± 18.98	AT-HIIT vs UC	10.00	0.46	19.53	*0.036*	0.33
	UC	69.76 ± 28.07	54.46 ± 34.16	80.81 ± 26.83						
Cognitive functioning	RT-HIIT	77.00 ± 26.05	78.19 ± 20.82	77.51 ± 27.95	RT-HIIT vs UC	3.47	− 5.45	12.39	1.000	0.23
	AT-HIIT	81.39 ± 20.60	79.72 ± 19.91	82.78 ± 18.90	AT-HIIT vs UC	7.30	− 1.69	16.28	0.153	0.30
	UC	78.78 ± 25.09	69.94 ± 27.42	73.28 ± 26.01						
Social functioning	RT-HIIT	64.98 ± 29.91	61.76 ± 48.79	81.22 ± 25.31	RT-HIIT vs UC	1.36	− 7.84	10.56	1.000	0.19
	AT-HIIT	72.91 ± 24.26	72.78 ± 24.92	89.45 ± 16.79	AT-HIIT vs UC	6.19	− 3.06	15.44	0.322	0.22
	UC	71.65 ± 29.63	62.20 ± 28.34	82.38 ± 26.64						
Fatigue	RT-HIIT	39.74 ± 29.88	37.58 ± 24.51	25.22 ± 22.86	RT-HIIT vs UC	− 7.39	− 16.99	2.21	0.194	− 0.44
	AT-HIIT	35.54 ± 23.28	38.52 ± 24.84	23.71 ± 20.63	AT-HIIT vs UC	− 9.98	− 19.66	− 0.30	*0.041*	− 0.40
	UC	34.08 ± 25.37	48.81 ± 25.58	31.87 ± 25.61						
Nausea and vomiting	RT-HIIT	12.87 ± 16.37	5.15 ± 10.49	2.38 ± 11.14	RT-HIIT vs UC	− 3.93	− 9.23	1.38	0.227	− 0.47
	AT-HIIT	12.99 ± 18.15	5.83 ± 12.21	2.50 ± 6.74	AT-HIIT vs UC	− 3.86	− 9.20	1.48	0.247	− 0.45
	UC	8.27 ± 17.16	8.04 ± 21.08	5.67 ± 16.00						
Pain	RT-HIIT	21.62 ± 24.82	21.32 ± 25.08	19.58 ± 24.60	RT-HIIT vs UC	− 6.39	− 16.46	3.67	0.379	− 0.29
	AT-HIIT	15.48 ± 22.40	16.95 ± 20.47	13.88 ± 19.68	AT-HIIT vs UC	− 9.23	− 19.36	0.89	0.086	− 0.29
	UC	17.32 ± 26.24	27.38 ± 29.89	22.65 ± 28.32						
Dyspnoea	RT-HIIT	25.11 ± 27.52	35.29 ± 29.86	20.43 ± 25.86	RT-HIIT vs UC	− 2.52	− 13.63	8.58	1.000	− 0.08
	AT-HIIT	22.25 ± 22.46	37.22 ± 28.19	19.42 ± 24.76	AT-HIIT vs UC	− 4.21	− 15.40	6.98	1.000	0.01
	UC	28.45 ± 25.70	44.05 ± 29.89	25.77 ± 24.14						
Insomnia	RT-HIIT	37.06 ± 30.00	31.37 ± 31.48	33.33 ± 31.68	RT-HIIT vs UC	3.01	− 9.90	15.92	1.000	0.04
	AT-HIIT	31.85 ± 25.58	26.11 ± 30.74	26.67 ± 26.62	AT-HIIT vs UC	− 1.38	− 14.38	11.61	1.000	0.01
	UC	33.32 ± 31.15	39.88 ± 35.63	28.31 ± 34.23						
Appetite loss	RT-HIIT	19.77 ± 29.00	13.73 ± 24.59	5.29 ± 13.66	RT-HIIT vs UC	− 4.01	− 11.46	3.42	0.579	− 0.37
	AT-HIIT	24.50 ± 26.70	20.00 ± 26.89	3.33 ± 11.81	AT-HIIT vs UC	− 8.38	− 15.94	− 0.82	*0.024*	− 0.66
	UC	13.64 ± 22.17	19.05 ± 27.60	8.81 ± 22.79						
Constipation	RT-HIIT	21.63 ± 27.96	10.78 ± 22.63	10.05 ± 19.52	RT-HIIT vs UC	0.40	− 6.95	7.75	1.000	− 0.08
	AT-HIIT	21.45 ± 27.09	12.22 ± 21.23	2.22 ± 8.38	AT-HIIT vs UC	− 8.42	− 15.81	− 1.03	*0.019*	− 0.37
	UC	18.70 ± 26.87	14.88 ± 24.55	9.43 ± 18.92						
Diarrhea	RT-HIIT	14.52 ± 22.58	8.82 ± 19.63	6.88 ± 14.86	RT-HIIT vs UC	2.65	− 4.55	9.84	1.000	0.13
	AT-HIIT	13.15 ± 22.87	18.89 ± 28.37	7.77 ± 18.78	AT-HIIT vs UC	2.77	− 4.48	10.02	1.000	0.23
	UC	15.78 ± 24.05	8.33 ± 17.12	5.03 ± 13.71						
Financial difficulties	RT-HIIT	21.73 ± 31.75	25.00 ± 35.21	17.46 ± 29.85	RT-HIIT vs UC	0.23	− 9.73	10.20	1.000	0.04
	AT-HIIT	16.54 ± 26.85	22.78 ± 34.98	12.23 ± 26.03	AT-HIIT vs UC	− 3.17	− 13.23	6.88	1.000	− 0.04
	UC	20.11 ± 31.95	19.05 ± 33.55	16.98 ± 32.44						

*ES* effect size, *EORTC-QLQ-C30* European Organization for research and cancer treatment quality of life questionnaire, *RT-HIIT* resistance and high-intensity interval training group, *AT-HIIT* moderate-intensity and high-intensity interval training group, *UC* usual care group

*Adjusted group differences based on all available data in the mixed-model analysis adjusted for baseline values; *p* < 0.05 is highlighted in italics

**Table 4:** Effects of the OptiTrain trial during chemotherapy on symptom burden at 12 months

						Adjusted group differences in mean change from baseline to 12 months				
		Baseline	16 weeks	12 months		Mean change	Lower bound 95% CI	Upper bound 95% CI	*p* value	ES
MSAS questionnaire		Mean ± SD	Mean ± SD	Mean ± SD						
Symptom burden	RT-HIIT	0.91 ± 0.72	0.77 ± 0.62	0.67 ± 0.63	RT-HIIT vs UC	− 0.14	− 0.35	0.08	0.394	− 0.41
	AT-HIIT	0.75 ± 0.58	0.66 ± 0.59	0.50 ± 0.47	AT-HIIT vs UC	− 0.27	− 0.49	− 0.06	*0.008*	− 0.46
	UC	0.70 ± 0.73	0.89 ± 0.67	0.72 ± 0.69						
Physical symptoms	RT-HIIT	0.74 ± 0.60	0.68 ± 0.57	0.36 ± 0.35	RT-HIIT vs UC	− 0.17	− 0.33	0.00	*0.049*	− 0.65
	AT-HIIT	0.67 ± 0.49	0.75 ± 0.59	0.34 ± 0.31	AT-HIIT vs UC	− 0.21	− 0.38	− 0.04	*0.008*	− 0.61
	UC	0.51 ± 0.56	0.77 ± 0.62	0.48 ± 0.52						
Psychological symptoms	RT-HIIT	0.97 ± 0.76	1.02 ± 0.77	0.87 ± 0.77	RT-HIIT vs UC	− 0.09	− 0.35	0.18	1.000	− 0.30
	AT-HIIT	0.81 ± 0.64	0.88 ± 0.73	0.69 ± 0.56	AT-HIIT vs UC	− 0.24	− 0.50	0.03	0.091	− 0.38
	UC	0.78 ± 0.77	1.11 ± 0.83	0.88 ± 0.80						
Total symptoms	RT-HIIT	0.74 ± 0.53	0.74 ± 0.50	0.52 ± 0.43	RT-HIIT vs UC	− 0.14	− 0.29	0.00	*0.044*	− 0.46
	AT-HIIT	0.65 ± 0.41	0.76 ± 0.51	0.44 ± 0.33	AT-HIIT vs UC	− 0.19	− 0.33	− 0.05	*0.003*	− 0.46
	UC	0.58 ± 0.50	0.85 ± 0.60	0.58 ± 0.50						

*ES* effect size, *MSAS* Memorial symptom assessment scale, *RT-HIIT* resistance and high-intensity interval training group, *AT-HIIT* moderate-intensity and high-intensity interval training group, *UC* usual care group

*Adjusted group differences based on all available data in the mixed-model analysis adjusted for baseline values; *p* < 0.05 is highlighted in italics

**Table 5:** Effects of the OptiTrain trial during chemotherapy on body mass, cardiorespiratory fitness, and muscle strength at 12 months

						Adjusted group differences in mean change from baseline to 12 months				
		Baseline	16 weeks	12 months		Mean change	Lower bound 95% CI	Upper bound 95% CI	*p* value	ES
		Mean ± SD	Mean ± SD	Mean ± SD						
Body mass (kg)	RT-HIIT	68.65 ± 11.34	69.47 ± 10.56	68.10 ± 9.64	RT-HIIT vs UC	− 1.55	− 3.56	0.47	0.195	− 0.22
	AT-HIIT	67.66 ± 13.00	67.34 ± 14.45	66.59 ± 10.89	AT-HIIT vs UC	− 2.28	− 4.29	− 0.26	*0.021*	− 0.24
	UC	69.07 ± 10.97	71.01 ± 11.66	70.93 ± 11.77						
Body mass index (kg/m^2^)	RT-HIIT	25.07 ± 4.28	25.38 ± 3.92	24.55 ± 3.19	RT-HIIT vs UC	− 0.56	− 1.06	− 0.06	*0.021*	− 0.38
	AT-HIIT	24.76 ± 4.41	24.64 ± 4.79	24.17 ± 3.59	AT-HIIT vs UC	− 0.91	− 1.41	− 0.42	*< 0.001*	− 0.39
	UC	24.96 ± 4.23	25.75 ± 4.52	26.05 ± 4.61						
Estimated VO2 peak (L/min)	RT-HIIT	2.25 ± 0.50	2.18 ± 0.57	2.51 ± 0.67	RT-HIIT vs UC	0.15	− 0.08	0.37	0.370	0.19
	AT-HIIT	2.10 ± 0.47	2.08 ± 0.49	2.33 ± 0.60	AT-HIIT vs UC	0.06	− 0.17	0.28	1.000	0.14
	UC	2.19 ± 0.53	1.93 ± 0.53	2.35 ± 0.65						
Estimated VO2 peak (mL/kg/min)	RT-HIIT	33.45 ± 7.91	31.70 ± 8.26	36.27 ± 9.11	RT-HIIT vs UC	3.20	− 0.17	6.57	0.068	0.40
	AT-HIIT	31.30 ± 6.65	31.36 ± 6.27	35.04 ± 9.90	AT-HIIT vs UC	3.08	− 0.26	6.43	0.081	0.56
	UC	32.40 ± 7.79	27.55 ± 6.64	32.09 ± 8.33						
Isometric mid-thigh pull (kg)	RT-HIIT	87.23 ± 29.55	100.24 ± 34.31	100.22 ± 29.29	RT-HIIT vs UC	15.90	6.11	25.70	*< 0.001*	0.73
	AT-HIIT	78.35 ± 25.11	88.26 ± 23.02	97.10 ± 30.26	AT-HIIT vs UC	17.66	7.64	27.67	*< 0.001*	1.03
	UC	89.32 ± 25.27	85.81 ± 25.96	82.05 ± 31.23						
Handgrip surgery side (kg)	RT-HIIT	28.40 ± 5.04	29.44 ± 5.27	29.86 ± 6.25	RT-HIIT vs UC	3.23	1.45	5.01	*< 0.001*	0.70
	AT-HIIT	28.44 ± 4.96	28.08 ± 5.29	29.93 ± 5.87	AT-HIIT vs UC	3.13	1.39	4.88	*< 0.001*	0.71
	UC	28.99 ± 6.16	27.72 ± 5.78	26.51 ± 7.32						
Handgrip non-surgery side (kg)	RT-HIIT	27.71 ± 4.93	28.39 ± 5.54	29.12 ± 6.70	RT-HIIT vs UC	2.87	0.85	4.89	*0.002*	0.57
	AT-HIIT	27.87 ± 5.44	27.41 ± 5.48	29.55 ± 6.47	AT-HIIT vs UC	2.71	0.73	4.69	*0.003*	0.59
	UC	28.47 ± 6.50	27.18 ± 6.33	26.62 ± 6.89						

*ES* effect size, *RT-HIIT* resistance and high-intensity interval training group, *AT-HIIT* moderate-intensity and high-intensity interval training group, *UC* usual care group

*Adjusted group differences based on all available data in the mixed-model analysis adjusted for baseline values; *p* < 0.05 is highlighted in italics

## Results

**Fig. 2:**
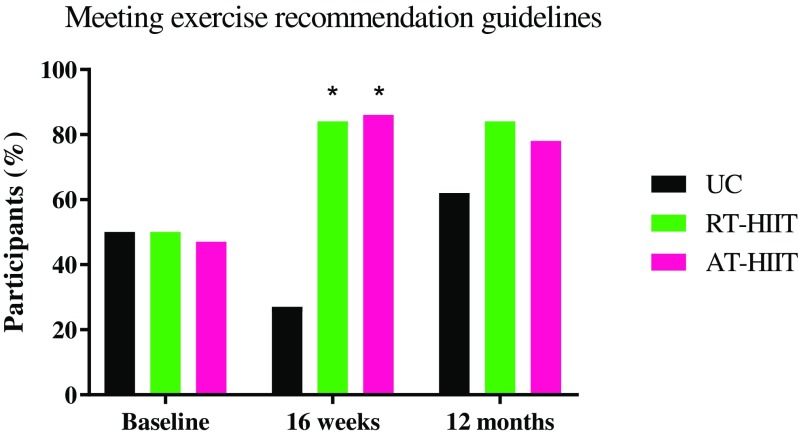
Percentage of each group reporting meeting exercise recommendation guidelines based on the single item questionnaire at baseline, 16 weeks, and 12 months post-baseline. RT-HIIT, resistance and high-intensity interval training group; AT-HIIT, moderate-intensity aerobic and high-intensity interval training group; UC, usual care group. *p < 0.05 vs UC

The time period for baseline and 12-month measurements ranged from March 2013 to July 2017. Attendance to the 16-week exercise intervention for participants in the RT-HIIT and AT-HIIT groups were 68 and 63%, respectively. At 12 months, 95% of the women who completed pre- and post-measurements filled out all questionnaires and 78% agreed to come back for in-clinic physiological reassessments (Fig. 1). There were no differences between intervention groups regarding participant characteristics (Table 1). Those that completed in-clinic assessments (muscle strength, cardiorespiratory fitness, and body mass) had significantly more moderate-vigorous PA min/day (objectively measured) at baseline (*p*=0.03) and had significantly higher attendance rates during the intervention period compared to those that were lost to follow-up or completed the survey only (*p*=0.005). However, there were no significant differences between the exercise groups and the UC group. Self-reported PA at baseline, 16 weeks and at 12 months are shown in Fig. 2. On average, 20% of the participants attended the motivational seminars (range = 11%–27%). Significant group × time (baseline to 12 months post-baseline) results are reported below.

### Primary outcome: cancer-related fatigue

**Fig. 3:**
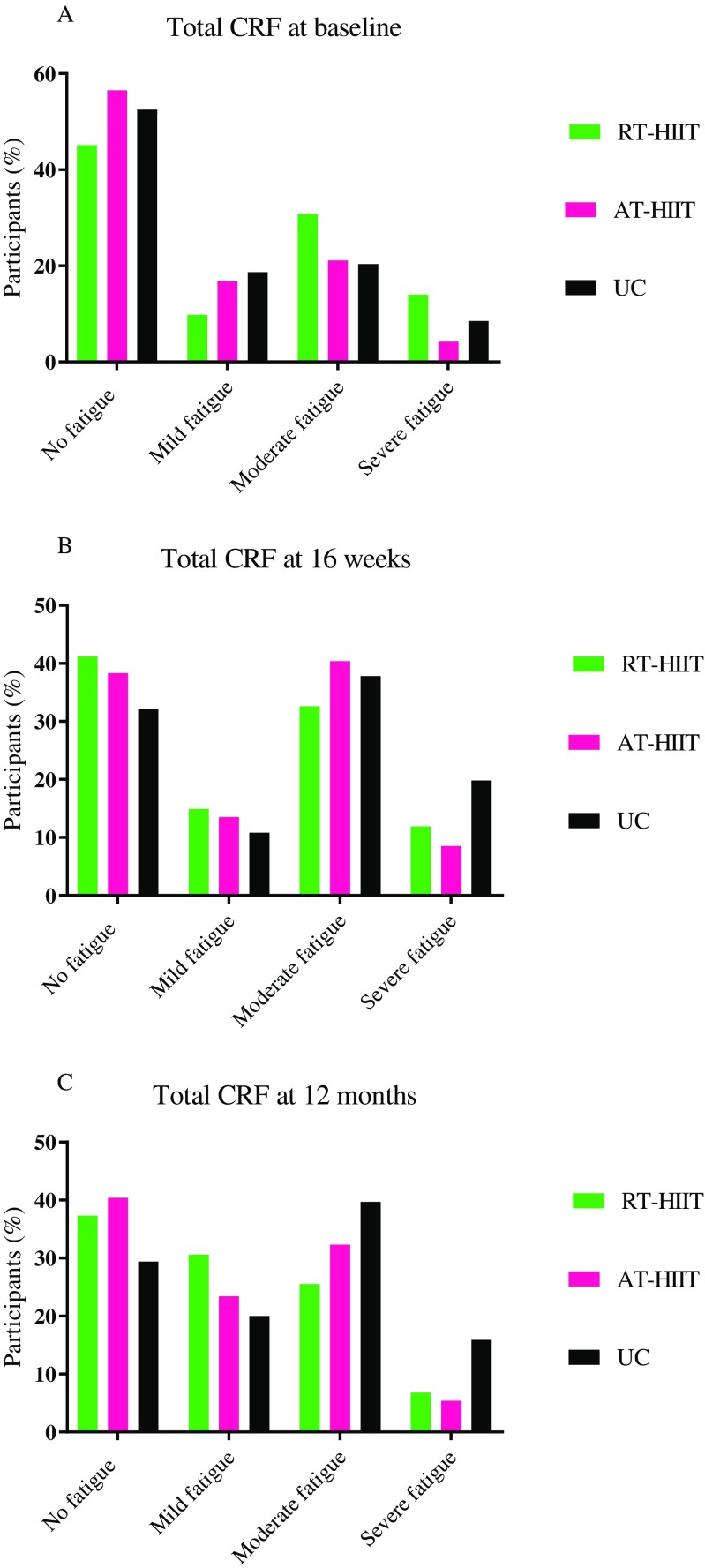
Total cancer-related fatigue levels divided into categories: no fatigue, mild fatigue (score > 0), moderate fatigue (score > 4), or severe fatigue (score > 7) at **a** baseline, **b** 16 weeks, and **c** 12 months post-baseline. RT-HIIT, resistance and high-intensity interval training group; AT-HIIT, moderate-intensity aerobic and high-intensity interval training group; UC, usual care group; CRF, cancer-related fatigue

Changes in CRF assessed by the Piper Fatigue Scale are shown in Table 2 and in Fig. 3. At 12 months, both RT-HIIT and AT-HIIT were superior to UC for total CRF (ES = − 0.34; ES = − 0.10), affective/emotional CRF (ES = − 0.60; ES = − 0.39), and behavior/daily life CRF (ES = − 0.76; ES = − 0.50). AT-HIIT counteracted increases in cognitive CRF (ES = − 0.13).

### Health-related quality of life

Changes in HRQoL from the EORTC-QLQ-C30 are shown in Table 3. For the functional subscales on the EORTC-QLQ-C30, role and emotional functioning favored AT-HIIT compared to UC (ES = 0.33; ES = 0.40). For the symptom subscale, AT-HIIT displayed favorable effects for fatigue (ES = − 0.40) and appetite loss (ES = − 0.66) compared to UC. AT-HIIT also had less constipation symptoms vs both RT-HIIT and UC (ES = − 0.28; ES = − 0.37).

### Symptom burden

Changes for symptoms as measured by the MSAS are shown in Table 4. At 12 months, both RT-HIIT and AT-HIIT reported significantly less total symptoms (ES = − 0.46; ES = − 0.46) and physical symptoms (ES = − 0.65; ES = − 0.61) compared to UC. Additionally, AT-HIIT reported lower symptom burden vs UC (ES = − 0.46).

### Muscle strength, cardiorespiratory fitness, and body mass

Changes in physiological outcomes are shown in Table 5. At 12 months, significant lower-limb strength gains were found for both exercise groups compared to UC (RT-HIIT, ES = 0.73; AT-HIIT, ES = 1.03). Significant handgrip strength gains were also shown for both RT-HIIT (surgery side, ES = 0.70; non-surgery side, ES = 0.57) and AT-HIIT (surgery side, ES = 0.71; non-surgery side, ES = 0.59) vs UC. AT-HIIT displayed significant reductions in body mass compared to UC (ES = − 0.24).

### Sick leave/return to work

At 12 months, the proportion of participants on more than half-time sick leave was significantly lower in the AT-HIIT group (5.9%) compared to the UC group (31.0%) (*p* = 0.006). In the RT-HIIT group 17.0% were on more than half-time sick leave. The difference remained significant between AT-HIIT (91%) and UC (69%) (*p* = 0.020) when assessing the proportion of participants that had partially or completely returned to work (i.e., from 100% sick leave to 50%, 25%, or 0% sick leave) compared to those that had not returned to work. In the RT-HIIT group, 82% had partially or completely returned to work.

### Associations between changes in CRF and changes in physiological outcomes

Significant negative associations on changes were found between lower limb muscle strength and CRF assessed by the EORTC-QLQ-C30 (*r* = − 0.25, *p* = 0.008), as well as CRF assessed by PFS (*r* = − 0.20, *p* = 0.035). Moreover, cardiorespiratory fitness was negatively associated with CRF measured by PFS (*r* = − 0.25, *p* = 0.005).

## Discussion

This is the first study to assess if resistance or aerobic training combined with high-intensity interval training (HIIT) during chemotherapy can induce benefits that are present several months after a breast cancer diagnosis. 1 year following the commencement of the OptiTrain exercise trial, we found beneficial effects of both RT-HIIT as well as AT-HIIT on fatigue, muscle strength, and symptoms. AT-HIIT also resulted in beneficial effects on body mass, symptom burden, role and emotional functioning, as well as lower sick leave rates compared to UC. These findings provide novel and important information with substantial positive consequences for breast cancer survivorship and clinical implications.

At 12 months, both RT-HIIT and AT-HIIT were beneficial to counteract CRF with moderate/large effect sizes found for RT-HIIT regarding behavioral/daily life CRF and emotional/affective CRF. Our findings are in contrast to previous follow-up trials consisting of combined resistance and aerobic training, which did not demonstrate sustained effects on fatigue at the 4–6 [12–15] or 12 month follow-up [28]. The discrepant results may be explained by the difference in information about PA given to the usual care group immediately following the interventions. In the current trial, the UC group is strictly treated as a control until the 5-year follow-up is completed. Moreover, the OptiTrain intervention is the first trial to provide physical activity prescriptions and motivational support to the exercise groups following the intervention, which may have resulted in inducing a behavioral change in the exercise groups as indicated by the self-reported PA data. Challenges to display beneficial effects of exercise training on cognitive and affective CRF has been evident in patients with breast cancer immediately following completion of chemotherapy [29]. Conversely, a trial conducted post-chemotherapy on breast cancer survivors assessing different exercise loads/intensities demonstrated that only the group performing high load resistance training combined with high-intensity continuous/interval training was able to counteract mental fatigue [30], and results from their 12-month follow-up study showed that the higher intensity group was superior to lower intensity exercise on role functioning [31]. It may be speculated that the HIIT component in the current trial may have played a role in inducing beneficial effects for cognitive CRF found for AT-HIIT, possibly through beneficial effects of HIIT on cerebral oxygenation [32] and/or improvements in information processing speed [33] as found in obese and elderly individuals, respectively. Despite low attendance at the motivational seminars, psychosocial factors that may have influenced outcomes for the exercise groups must be taken into account given that the UC group did not receive comparable motivational support. However, it must be noted that this finding may not be clinically meaningful due to the low effect size (ES = 0.13).

It remains unknown whether fatigue displayed during survivorship is due to cancer treatment, inactivity, or underlying symptoms/co-morbidities. Over the 16-week intervention, we found a significant negative association between changes in CRF and in lower-limb muscle strength [10]. The same association was found at 12 months, suggesting muscle weakness as a factor underlying CRF. Our findings are in line with a cross-sectional study that demonstrated poor lower-extremity muscle strength as one of the predictors of fatigue in long-term breast cancer survivors [34].

The effects of exercise on sick leave/return to work has received increased attention in patients with cancer. In line with our findings, an exercise trial conducted after completion of chemotherapy demonstrated that those who had performed high-intensity exercise had an increased ability to work [35]. Physical and cognitive fatigue/impairments have been shown to affect work capacity, and fatigue 6 months after chemotherapy predicted a longer sick leave [36]. Both exercise groups counteracted fatigue at 12 months, and it may be speculated that the superior effects found for AT-HIIT on sick leave/return to work may be due to the ability to counteract cognitive fatigue compared to the UC group. In addition to experiencing increased cognitive fatigue, although no significant differences were found between groups, the UC group showed lower levels for psychological symptoms and cognitive functioning on the MSAS and EORTC-QLQ-C30 subscales, while other symptoms and quality of life functional-related aspects had returned to baseline levels. This is in line with a recent longitudinal exercise trial for patients with breast cancer showing that cognitive functioning was still impaired while quality of life-related functions improved over time since chemotherapy [37].

The OptiTrain trial is the first to report on exercise effects on symptoms using a symptom-specific scale. The exercise interventions were effective in relieving, and even showing improvements, for multiple dimensions of symptoms at the follow-up. A previous study showed that more than one in four cancer survivors had high symptom burden 1 year post diagnosis [38], emphasizing the important role of exercise interventions during chemotherapy in managing persistent symptoms.

Both exercise groups displayed significant strength gains with moderate to large effect sizes. The RT-HIIT group was able to maintain the muscle strength gained during chemotherapy into survivorship, while the AT-HIIT group gained muscle strength after chemotherapy to similar levels as the RT-HIIT group at the follow-up. It may be speculated that since the AT-HIIT group performed sessions at the same time as RT-HIIT, the AT-HIIT group may have been inspired and incorporated resistance exercise training in their activities after completion of the intervention. A study that assessed PA attendance of breast cancer survivors up to 12 months, after a combined resistance and aerobic exercise intervention, showed that strength exercise was reported to be the most common form of exercise [39]. Nevertheless, few previous trials conducted during chemotherapy have found differences between exercise and control groups at the follow-up [13–15]. A persistent decline in muscle strength is associated with a decline in overall health and quality of life among breast cancer survivors [40] and is a strong independent predictor of all-cause mortality in healthy elderly [41]. Therefore, the implementation of high-load resistance exercise during chemotherapy is of major importance as well as motivational support to maintain/implement resistance training after completion of chemotherapy.

Previous findings have shown an average weight gain of 2 kg in women with breast cancer 1 year after chemotherapy and difficulties to return to pre-diagnosis body weight [42]. Emerging evidence indicate that exercise has a negligible impact on weight loss in healthy individuals [43]. Whether exercise interventions can limit the increase in body mass in patients with breast cancer is largely unknown. Here, the women who had performed exercise during chemotherapy displayed a reduced body mass at the follow-up, with only the AT-HIIT group reaching the significance level compared to the UC group that instead gained weight. Previous trials were unable to show any effects of combined resistance and endurance training on follow-up body weight [14, 17] or body composition [15], possibly due to a sustained muscle mass as a result of resistance training.

Given that knowledge and skills of conducting exercise has been shown to be a predictor of exercise adherence after a cancer treatment [44], we speculate that participation in the OptiTrain trial as well as PA prescriptions facilitated the engagement in exercise training after completion of chemotherapy. Barriers to engage in physical exercise, such as lack of knowledge regarding exercise [45], as well as increased fatigue [4] may have been more difficult to overcome in the control group.

Strengths of this study include the limited loss to follow-up, a high response rate, and in-clinic measurements of objective muscle strength, cardiorespiratory fitness, and body mass. Moreover, treating the UC group as a control group throughout the duration of the follow-up period facilitates the interpretation of the exercise-trial effects. Limitations include a more active sample that completed physiological in-clinic assessments, which introduces a potential selection bias. However, since this also applied to the UC group, it should not influence the between groups comparisons. Other limitations include not measuring PA objectively at all time points, and not having more detailed information regarding type and intensity of exercise performed, which limits our conclusions on whether any change in PA behavior took place. However, objective PA is currently being measured at the 2-year assessment and will provide valuable insights into activity levels across all groups.

## Conclusion

Both RT-HIIT and AT-HIIT displayed beneficial effects on cancer-related fatigue, symptoms, and muscle strength, 12 months following the commencement of chemotherapy. Additionally, favorable changes in body mass and return to work were observed for the participants that had been part of the AT-HIIT group during chemotherapy. Importantly, we found moderate to large effect sizes for several outcomes, including fatigue. These findings show that participating in a supervised exercise program for women with breast cancer, with support to maintain physical exercise, can induce long-term benefits. These benefits are not limited to the individual, but also leads to societal benefits with reduced costs associated with prolonged sick leave. Strategies are needed to support patients to exercise both independently and with supervision, throughout the cancer survivorship continuum. It is important to create an awareness of the value of exercise and to provide both patients and health professionals with information on available exercise programs, resources, and qualified exercise specialists. Cost-effectiveness studies on supervised exercise programs for patients with breast cancer are warranted. Future research should also focus on evaluating the safety and effectiveness of innovative exercise approaches such as artificial intelligence exercise solutions that are easy to administer and carry out without supervision.
